# Associations of fundamental motor skill competence, isometric plank, and modified pull-ups in 5-year old children: An observational analysis of 2012 NHANES NYFS

**DOI:** 10.1371/journal.pone.0276842

**Published:** 2022-10-27

**Authors:** E. Andrew Pitchford, Willie Leung, E. Kipling Webster

**Affiliations:** 1 Department of Kinesiology, Iowa State University, Ames, IA, United States of America; 2 Department of Health Sciences and Human Performance, University of Tampa, Tampa, FL, United States of America; 3 Institute of Public and Preventive Health, Augusta University, Augusta, GA, United States of America; Iran University of Medical Sciences, ISLAMIC REPUBLIC OF IRAN

## Abstract

There are purported relationships between fundamental motor skills (FMS), health-related physical fitness, physical activity, and obesity among children. The purpose of this observational, secondary data analysis was to further examine these associations in children from the 2012 National Youth Fitness Survey (NYFS). 121 five-year old children (51% female) from the NYFS database completed the Test of Gross Motor Development, 2nd edition (TGMD-2), muscular fitness measures of plank and modified pull-ups, and weight status based on body mass index (BMI) percentile. Significant positive correlations were identified between TGMD-2 scores and both pull-ups and plank. Linear regression models, controlling for sex, weight status, and Hispanic ethnicity also identified both modified pull-ups completed and plank time as significant predictors of TGMD-2 total raw score. No demographic factors were significant factors in any of the models. This secondary data analysis identified associations between FMS and health-related physical fitness (i.e., muscular fitness), with both modified pull-ups and plank performance, in five-year old children. Results underscored the importance of different facets of health-related physical fitness contributing significantly to FMS performance and the need for more work related to physical fitness in preschool-age children.

## Introduction

Fundamental motor skills (FMS) are basic, essential movements that are developed in early childhood [1] and are combined into more complex patterns in order to participate in more sustained physical activities [2]. These FMS are gross motor patterns that involve large muscle groups, particularly locomotor skills where one moves through space and object control skills where you are manipulating or projecting objects [3].

Over a decade ago, a conceptual model was proposed hypothesizing the central role FMS plays alongside physical activity in a reciprocal and dynamic relationship which contributes to children’s health [4]. Physical fitness and perceived motor competence played a mediating role in this contribution towards weight status [4]. Robinson and colleagues [5] conducted a systematic review that provided strong evidence for the proposed model. Positive relationships of FMS with physical activity and physical fitness, and an inverse relationship with weight were reported. Further, perceived motor competence and physical fitness can mediate the primary relationship between motor competence and physical activity, although the strength or direction of these relationships can change over time [5]. A follow-up systematic review elaborated on the mediational, longitudinal, and experimental evidence since Robinson et al.’s review [5] and reported mixed results in terms of the role motor competence plays in the health trajectory model, but found strong support for the relationship between motor competence and physical fitness [6].

A plethora of research is available on the relationships between FMS and physical activity or physical fitness. Specifically, more physically fit children [7–10] tend to have higher FMS. Previous work has also shown that different types of FMS (i.e., locomotor and object control) may be better predictors of certain aspects of overall health. For instance, FMS in early childhood predicted physical fitness approximately six years later, where children who had better object control skills were more physically fit as adolescents [11]. Conversely, performance on a cardiorespiratory test in early childhood predicted motor competence performance three years later [12]. In addition, physical fitness has also been shown to mediate the relationship between physical activity and motor competence [13], which emphasizes the important role physical fitness plays in relation to overall motor competence. Cattuzzo [7], reviewed the literature on FMS and physical fitness in children and found strong evidence for a positive association between FMS competence and body weight status, cardiorespiratory fitness, and muscular fitness. These findings were extended through a meta-analysis from Utesch [10] which found moderate to large associations between FMS competence and physical fitness in children, highlighted by strengthened relationships with increasing age. Similarly, a cross-sectional investigation of children 4–13 years of age found that the association between FMS, specifically object control skills, and physical fitness increased with age [14]. In many of these previous investigations, cardiorespiratory fitness is generally the primary component of health-related fitness that is used to assess the relationship with motor competence, with only a few studies also including flexibility and muscular endurance or strength [6].

Physical fitness is an important, multi-faceted indicator of body function and is associated with health outcomes in youth [15,16], encompassing a variety of factors including cardiorespiratory fitness, muscular fitness (i.e., muscular strength and endurance), flexibility, and body composition [15]. A variety of positive associations have been shown between physical fitness in adolescence and health outcomes as young adults, most notably cardiovascular disease risk [16]. Burgeoning evidence of this relationship is available for younger children, but is limited due to the challenges associated with measuring physical fitness and sparse comprehensive assessments validated in this population [15].

For the 2012 National Health and Nutrition Examination Survey (NHANES) National Youth Fitness Survey (NYFS) the Test of Gross Motor Development– 2^nd^ edition (TGMD-2) [17], was adopted to further examine how FMS impacted other health factors in young children, 3–5 years of age [18]. Several studies are available that have utilized this cohort to examine several factors, including: the breakdown of FMS in a nationally representative sample [19], FMS and free-living physical activity [20], FMS and organized physical activity [21], FMS and outdoor physical activity [22], FMS and plank performance [23], FMS and body fat percentage [24], familial and socioeconomic influences on FMS [25], the relationship between FMS, plank performance, and BMI by Hispanic ethnicity [26], and differences in FMS in children with disabilities [27]. These studies on FMS utilizing TGMD-2 data from the 2012 NHANES NYFS add to the literature by leveraging the national sample of NHANES to examine relevant relationships.

Two studies, in particular, were examinations of the relationship between FMS and musculoskeletal fitness [23,26]. Both used the isometric plank exercise assessment to reflect musculoskeletal fitness. Frith et al. [23] reported a significant positive relationship between plank performance and FMS for the standardized TGMD-2 gross motor quotient, locomotor subscale, and object control subscale after controlling for physical activity, age, sex, race/ethnicity, asthma, and weight status. Zhang et al. [26] examined similar relationships between children of Hispanic and non-Hispanic ethnicities. Among Hispanic children, plank performance was associated with locomotor skills, whereas the plank was associated with object control skills among non-Hispanic children after controlling for physical activity, sedentary behavior, sex, and age [26]. However, the Institute of Medicine (IOM) has recommended that no single fitness test is adequate to describe musculoskeletal fitness, due to the multiple components within the construct (i.e., strength, endurance, power) and the variety of muscle groups for which fitness can be assessed [15]. Thus, these analyses from Frith et al. [23] and Zhang et al. [26] may be limited by the sole focus on isometric plank performance. There are additional musculoskeletal fitness measures available in the 2012 NHANES NYFS that could be used to further examine this relationship. Through examination of multiple relationships of FMS and musculoskeletal fitness, we may be able to ascertain which fitness tests are the most powerful correlates of FMS competency in children.

The 2012 NHANES NYFS includes assessments of TGMD-2 for children 3–5 years, isometric plank for children 3–16 years, and modified pull-ups for children 5–16 years. Thus, if these data were to be analyzed specifically for five-year-old children, the relationship between FMS and multiple indicators of musculoskeletal fitness can be examined. Therefore, the purpose of this observational study was to utilize the sample of five-year-old children from the 2012 NHANES NYFS to examine the relationships among FMS and muscular fitness with both isometric plank and modified pull-up performance.

## Materials and methods

### Design and participants

This study is a secondary analysis of cross-sectional data from the 2012 NHANES NYFS. The 2012 NHANES NYFS was a multi-stage probability sample of the civilian non-institutionalized resident population of the United States with sampling conducted by sex and age bands of 3–5, 6–11, and 12–15 years [28]. Measures included survey interviews and a physical examination conducted by the NHANES NYFS staff of nurse practitioners and examiners with expertise in exercise physiology, nutrition, kinesiology. All procedures of the 2012 NHANES NYFS were reviewed and approved by the National Center for Health Statistics Ethics Review Board. Written informed consent to participate was provided by the participant’s legal guardian [28].

In the 2012 NHANES NYFS dataset, among 3–5 year old children, 368 completed the screening and 352 completed the physical examination [28]. Participants for this secondary analysis were limited to five-year old children. Only five-year old children were included in the current analysis because this was the only age group that completed assessment of TGMD-2 for FMS competency and both plank and modified pull-ups for muscular fitness. A total of 123 5-year-old children participated in the survey. Participants with complete data on the following measures were included in the analysis. Two participants were excluded for missing data, resulting in a final sample of 121 five-year old children for all analyses.

### Study variables

#### Fundamental motor skills

The Test of Gross Motor Development-2 was used to assess FMS competency, including locomotor (run, gallop, hop, leap, jump, and slide) and object control skills (strike, dribble, catch, kick, throw, roll). All procedures were standardized, following both the TMGD-2 manual [17] and NYFS study protocol [18] and were performed by trained and experienced staff under the consultation of the TGMD-2 developer (Dale A. Ulrich). The TGMD-2 is a popular assessment of FMS competency in children and has appropriate psychometric evidence for validity and reliability [17]. TGMD-2 data used included raw scores from each of the 12 skills, raw and standardized scores for the locomotor and object control subscales, and the composite Gross Motor Quotient (GMQ). Standardized scores reflect a norm-referenced evaluation of performance compared to children in the United States based on age (in six month intervals) and sex (object control only) [17]. Subscale standard scores have a mean score of 10 with a standard deviation of 3, while the GMQ has a mean score of 100 with a standard deviation of 15. Thus, standardized scores between 6 and 14 on each subscale, and between 80 and 120 for GMQ, can be interpreted as “average” performance. Scores above and below that range can be interpreted as “superior” and “poor” performance, respectively. The descriptive terminology is based on the TGMD-2 manual [17]. Children were classified into poor, average, and superior categories for each subscale and GMQ based on standardized scores to describe the sample. Raw TGMD-2 scores for each subscale were used to examine associative relationships with musculoskeletal fitness to allow for direct effect of sex to be included in models without influence of standardization.

#### Muscular fitness

The plank was used as an assessment of isometric core muscular endurance. Plank procedures followed a standardized protocol [29]. Each participant was instructed to form a plank position (i.e., prone position, only hands, forearms and toes touch floor, back straight, tighten stomach and thigh muscles) and hold the pose for as long as possible. The trial ended when the participant could no longer hold the proper pose (e.g., arching back, dipping hips) or elected to stop. Each participant was given a practice test to check for correct position prior to the test trial. The plank score was recorded as the number of second the participant held the plank pose [29]. Consistent with previous analyses of these data [26], a participant with a “completed” status code, but a comment of “could not obtain” were treated as a plank of 0 seconds. Initially, data between the “could not obtain” group were treated as missing and were compared to those who completed the task to determine if any significant differences were present; the exclusion of this data did not alter the interpretation of the findings and were therefore maintained. Any participant with a “not done” status code were treated as missing data and were excluded.

The modified pull-up was used as an assessment of upper body muscular strength. The procedures for the modified pull-up were based on the *FitnessGram* [30] and followed a standardized protocol [31]. The modified pull-up used an adjustable horizontal bar apparatus with an 8 inch strap hanging below. The participant began in a supine position on the floor with arms extended. The horizontal bar was set at a height of 2 inches beyond the outstretched hands. To begin, the participant clasped the horizontal bar with an overhand grip (i.e., palms face away from body) and lifted the body so that only heels touch the ground. Each repetition involved flexing the arms until the chest touched the 8-inch strap and then lowering the body back to the starting position; keeping the body straight throughout. The trial ended when the participant could no longer maintain proper form, paused for 2 or more seconds, or elected to stop. Any children with a “not done” status code on the modified pull-up test were treated as missing data and were excluded.

#### Weight status

Body mass index (BMI) percentile was used to determine overweight/obesity status in the NYFS protocol [32]. Each participant was measured for height and weight to calculate BMI (kg/m^2^). Standing height was measured using a SECA 217 stadiometer to the nearest 0.1 centimeters. The participant was instructed to stand barefoot with both feet flat, heels together and toes apart (60° angle), four points of contact on the backboard (i.e., heels, buttocks, shoulder blades, head), and head in a horizontal position. Weight was measured to the nearest 0.01 kilograms with participants barefoot with light clothing using a SECA 869 digital scale. BMI was calculated from these measurements: weight (kg) / height squared (m^2^) and were further evaluated using the 2000 CDC sex-specific BMI-for-age growth charts for BMI [33]. For classification of weight status, children were identified as overweight/obese if the BMI percentile was ≥ 85 percentile.

#### Demographic survey

Participant demographics, including sex (female, male) and Hispanic origin (Hispanic, non-Hispanic) were collected from a demographic questionnaire completed by a proxy respondent (e.g., parents or legal guardians). Age (in years) was recorded at the time of the physical exam.

### Statistical analysis

All data were downloaded from the National Center for Health Statistics website and combined. Statistical analyses were conducted using R (Vienna, Austria), with the “survey” package, accounting for the 2012 NHANES NYFS survey design with sampling weights, primary sampling unit indicators, and stratum variables [28]. Descriptive statistics were calculated as mean ± standard error and proportion with 95% confidence interval (CI) for continuous and categorical variables, respectively. TGMD-2 raw scores were used to reflect FMS competency for all associative and inferential analyses. Group differences were examined with independent-group t-tests to examine differences in FMS competency and muscular fitness based on sex, weight status, and Hispanic ethnicity. Bivariate correlations were used to examine associative relationships between outcomes and were interpreted based on magnitude ranges proposed by Cohen [34]. Finally, linear regression was used to further examine the associative relationship between FMS competency and muscular fitness with the covariates of sex, weight status, and Hispanic origin. TGMD-2 raw scores were the dependent variable for all regression models, with separate analyses for plank and modified pull-ups as independent variable. Each analysis included multiple models to account for independent covariates. Alpha level was set at .05 for all analyses.

## Results

121 five-year old children (5.09 ± 0.02 years; 51.40% female) were selected from the NYFS national sample based on complete data. **Table 1** summarizes the descriptive statistics for sample demographics, FMS competence, and muscular fitness. Measures of muscular fitness were highly variable for both modified pull-ups (2.18 ± 0.37 repetitions) and plank (24.06 ± 2.25 seconds). Of note, 35.30% (95% CI: 25.80, 46.00) of the sample were not able to complete a single modified pull-up (i.e., score of 0 repetitions).

**Table 1:** Characteristics of 5-year-old children from the 2012 NYFS.

		Unweighted Sample Size (*n* = 121)	Weighted Mean ± SE or Weighted Proportion (95%CI)
Sex	Female	61	51.40 (40.90, 62.00)
	Male	60	48.60 (38.30, 59.00)
Age in years			5.09 ± .02
Hispanic	Hispanic	48	29.40 (14.90, 50.00)
	Non-Hispanic	73	70.60 (50.10, 85.00)
BMI (kg/m^2^)			16.36 ± .18
BMI percentile			61.55 ± 2.50
Weight status	< 85%ile	85	68.32 (60.40, 75.00)
	≥ 85%ile	36	31.68 (24.70, 40.00)
TGMD-2 standardized scores	Gross Motor Quotient		95.90 ± 1.54
	Locomotor standard score		10.02 ± .29
	Object Control standard score		8.60 ± .30
TGMD-2 raw scores	Total raw score		60.30 ± 1.44
	Locomotor raw subscale		33.63 ± .80
	Object Control raw subscale		26.63 ± .90
TGMD-2 skill scores	Run		7.58 ± .09
	Gallop		5.69 ± .27
	Hop		6.56 ± .44
	Leap		3.69 ± .22
	Jump		3.56 ± .21
	Slide		6.70 ± .26
	Two-hand Strike		6.36 ± .26
	Dribble		3.64 ± .25
	Catch		4.18 ± .17
	Kick		6.08 ± .20
	Overhand Throw		1.42 ± .22
	Underhand Roll		4.96 ± .24
Muscular fitness	Plank (seconds)		24.06 ± 2.25
	Modified Pull-up (count)		2.18 ± .37

*Note*. Descriptive statistics are presented as Mean ± Standard Error (SE) or Proportion (95% Confidence Interval [CI]). BMI = body mass index, 85%ile = BMI percentile based on CDC growth charts, TGMD-2 = Test of Gross Motor Development, 2^nd^ edition.

Based on the TGMD-2 normative scales for average performance on the GMQ (100) and each subscale (10), average FMS competency scores of the sample were slightly lower than normal for the GMQ (95.90 ± 1.54) and object control skills (8.60 ± .30), but normal for locomotor skills (10.02 ± .29). GMQ scores indicated most children had average FMS competence (75.20%, 95% CI [62.20, 84.83]), while 16.11% (95% CI [9.33, 26.00) were poor and 8.68% (95% CI [3.79, 18.64]) were superior. Similar distributions were also observed for the locomotor scores: poor (10.30%; 95% CI [5.38, 18.83), average (79.50%; 95% CI [67.71, 87.73), and superior (10.2%; 95% CI [5.20, 19.11]) and object control subscales: poor (12.05%; 95% CI [7.04, 19.85]), average (88.00%; 95% CI [80.15, 92.96]) average, and no superior scores (0%).

Differences in FMS and muscular fitness based on sex, weight status, and Hispanic ethnicity are reported in **Table 2**. Multiple significant differences in modified pull-up performance were observed. Non-Hispanic children completed significantly more pull-ups (2.61 ± .33) than Hispanic children (1.11 ± .38, *p* = .006). Normal weight children (< 85 BMI %ile) also completed more pull-ups (2.50 ± .46) than overweight/obese (≥85 BMI %ile) children (1.51 ± .29, *p* = .031). No demographic differences were observed for total TGMD-2 raw score or plank performance.

**Table 2:** Average TGMD-2 raw score, number of pull up completed, duration of plank of five-year old children stratified by sex, weight status, and Hispanic origin.

	TGMD-2 raw score				Modified Pull-up (count)				Plank (seconds)			
	Average	SE	t	*p*	Average	SE	t	*p*	Average	SE	t	*p*
**Total** (*n* = 121)	60.30	1.45			2.18	.37			24.06	2.25		
**Sex**			-.19	.855			.40	.693			.69	.501
Male (*n* = 60)	60.56	1.75			2.08	.47			22.33	3.15		
Female (*n* = 61)	60.02	2.34			2.28	.41			25.86	3.62		
**Weight status**			-.67	.513			-2.42	**.031** *			-.03	.977
< 85%ile (*n* = 85)	60.95	1.39			2.50	.46			24.10	2.81		
≥ 85%ile (*n* = 36)	58.95	2.84			1.51	.29			23.96	3.65		
**Hispanic**			-.09	.930			-3.27	**.006** *			.08	.934
Hispanic (*n* = 48)	60.10	2.46			1.11	.38			24.39	5.13		
Non-Hispanic (*n* = 73)	60.38	1.82			2.61	.33			23.92	2.28		

*Note*. Descriptive statistics are presented as weighted mean ± standard error (SE). 85%ile = weight status based on BMI percentile from CDC growth charts, TGMD-2 = Test of Gross Motor Development, 2^nd^ edition, t = t-value of group comparisons (i.e., male and female, below and above 85%ile, and Hispanic and non-Hispanic)

*p-value < .05.

Pearson bivariate correlation was employed to examine associative relationships between FMS competence and muscular fitness. Statistically significant correlations were identified between total TGMD-2 raw score and both modified pull-ups (*r* = .34, SE = .06, *p* < .001) and plank (*r* = .50, SE = .05, *p* < .001). Based on the magnitude of association [34], these relationships can be interpreted as medium to large, respectively. The plank and modified pull-ups measures were also significantly correlated with each other with medium magnitude (*r* = .37, SE = .09, *p* < .001).

Finally, linear regression models were employed to further examine associative relationships between FMS competence and muscular fitness while controlling for demographic variables. Results with modified pull-ups (**Table 3**) and plank performance (**Table 4**) are presented separately. Modified pull-ups had a significant relationship with FMS competency (β = 1.82, *p* = .016) and the full model explained 12.6% of TGMD-2 raw score variance (*F*(8,107) = 12.39, *p* = .073). There were no significant covariates in this model. The plank also exhibited a significant relationship with FMS competency (β = .31, *p* < .001), with the model explaining 25.5% of the variance in TGMD-2 raw score (*F*(8,107) = 38.06, *p* < .001). Again, there were no significant covariates in this model.

**Table 3:** Linear regression of TGMD-2 raw scores and modified pull-ups completed by five-year old children.

	Model 1^a^		Model 2^b^		Model 3^c^		Model 4^d^		Model 5^e^	
	*β*	*p*	*β*	*p*	*β*	*p*	*β*	*p*	*β*	*p*
**Modified Pull-up** (count)	1.73	**.004** *	1.73	**.005** *	1.72	**.006** *	1.82	**.011** *	1.82	**.016** *
**Sex**										
Male [ref]			1						1	
Female			-.89	.751					-.89	.752
**Weight status**										
< 85%ile [ref]					1				1	
≥ 85%ile					-.31	.919			-.14	.963
**Hispanic**										
Hispanic							2.51	.498	2.48	.495
Non-Hispanic [ref]							1		1	
**R** ^**2**^	.119		.120		.119		.125		.126	
**AIC**	7.819		10.198		10.259		11.252		15.952	
**BIC**	21083.80		21083.55		21083.32		21082.73		21081.53	

Note.

^a^Coefficient from linear regression model consisted of number of pull ups completed.

^b^Coefficient from linear regression model consisted of number of pull ups completed and sex (male [ref] and female).

^c^Coefficient from linear regression model consisted of number of pull ups completed and weight status (< 85%ile [ref], ≥ 85%ile based on CDC growth charts).

^d^Coefficient from linear regression model consisted of number of pull ups completed and Hispanic origin (Hispanic and non-Hispanic [ref]).

^e^Coefficient from linear regression model consisted of number of pull ups completed and covariates of sex (male [ref] and female), weight status (< 85%ile [ref], ≥ 85%ile), and Hispanic origin (Hispanic and non-Hispanic [ref]). *β*, regression coefficient.

*p-value < .05.

**Table 4:** Linear regression of TGMD-2 raw scores and plank performance by five-year old children.

	Model 1^a^		Model 2^b^		Model 3^c^		Model 4^d^		Model 5^e^	
	*β*	*p*	*β*	*p*	*β*	*p*	*β*	*p*	*β*	*p*
**Plank** (seconds)	.31	**< .001** *	.31	**< .001** *	.31	**< .001** *	.31	**< .001** *	.31	**< .001** *
**Sex**										
Male [ref]			1						1	
Female			-2.00	.329					-2.06	.310
**Weight status**										
< 85%ile [ref]					1				1	
≥ 85%ile					-2.13	.363			-2.24	.345
**Hispanic**										
Hispanic							.08	.975	-.01	.997
Non-Hispanic [ref]							1		1	
**R** ^**2**^	.245		.250		.250		.245		.255	
**AIC**	8.929		10.439		10.710		11.172		14.270	
**BIC**	17697.25		17697.17		17697.13		17696.94		17695.76	

Note.

^a^Coefficient from linear regression model consisted of duration of plank in seconds.

^b^Coefficient from linear regression model consisted of duration of plank in seconds and sex (male [ref] and female).

^c^Coefficient from linear regression model consisted of duration of plank in seconds and weight status (< 85%ile [ref], ≥ 85%ile based on CDC growth charts).

^d^Coefficient from linear regression model consisted of duration of plank in seconds and Hispanic origin (Hispanic and non-Hispanic [ref]).

^e^Coefficient from linear regression model consisted of duration of plank in seconds and covariates of sex (male [ref] and female), weight status (< 85%ile [ref], ≥ 85%ile), and Hispanic origin (Hispanic and non-Hispanic [ref]). *β*, regression coefficient.

*p-value < .05.

## Discussion

The purpose of this study was to utilize the 2012 NHANES NYFS to examine the relationships among FMS, muscular fitness, and body composition within a sample of five-year old children. Statistically significant relationships between FMS and indicators of muscular fitness, including plank and modified pull-ups, were identified. The primary finding of the study was that FMS, as measured by the TGMD-2, was significantly correlated with muscular fitness on both plank and modified pull-up exercises, but not body composition.

Based on previous research, a positive associative relationship between FMS competence and measures of physical fitness is to be expected during childhood, increasing in strength with age [4,5]. The current analysis provides additional evidence for this relationship as both plank and modified pull-ups, measures of core and upper-body muscular endurance, respectively [28], were significantly associated with competence in FMS as measured by the TGMD-2. Previous studies utilizing the 2012 NHANES NYFS data in 3–5 year old children have also reported significant relationships between plank performance and FMS competence [23,26]. To our knowledge, this is the first study to include an analysis of modified pull-ups with FMS from 2012 NHANES NYFS. These findings are consistent with the majority of the published literature. Systematic reviews and meta-analyses on the connection between FMS and physical fitness have found strong evidence to support a positive relationship during childhood [6,7,10]. The breadth of evidence specific to preschoolers is limited, but still suggests that greater motor competence is associated with factors of physical fitness, and vice versa. For example, Oberer [35] found small to medium associations between the jumping sideways skill from the *Körperkoordinationstest für Kinder* (KTK) and both the six minute run (*r* = .24) and standing long jump (*r* = .44), in a sample of children 5–7 years old. Stodden et al. [14] also found medium correlations between muscular fitness and cardiorespiratory endurance, measured by pushups, curl-ups, grip strength and the PACER test, on kicking (*r* = .38) and jumping (*r* = .55) skills, but not throwing (*r* = .23), in a sample of 4–6 year old children. Lastly, Sigmundsson [36] found a significant association between FMS scores and physical fitness (r = -.61) in a sample of 4–6 year old children. The inverse relationship was due to lower FMS scores, as measured by the MABC-2, indicating better motor competence [36]. The present study, in concert with other NHANES investigations [23,26], furthers this body of evidence by identifying that the positive associations between FMS competency and physical fitness are also significant for process-oriented motor assessments (i.e., TGMD-2) in early childhood for two muscular fitness tests.

No significant relationships between body weight status and FMS competence were observed within the sample of five-year old. Kit [19] previously found that there were no differences in either locomotor or object control scores across obese, overweight, and normal weight status groups within the 2012 NHANES NYFS. However, the majority of the literature is consistent on a relationship between FMS competence and body composition in childhood. Cattuzzo [7] reported that of the 33 studies identified in their review, 82% reported an inverse relationship between weight status and FMS competence. Additionally, Cheng [37] showed that obesity at the age of 5 was associated with poorer FMS, compared to their normal or overweight peers, and obesity was significantly associated with a decline in FMS skills over 5 years. Future work is needed to better understand this relationship as morphological constraints may impact motor skills depending on whether a task requires a change in one’s center of mass rather than force production or fine motor coordination [38].

While body composition was not significantly correlated with FMS competency, a significant negative association between modified pull-ups and body weight status was observed. This association is logical as greater adiposity will make the task of repeated modified pull-ups more difficult. As the modification of the pull-up task to an inverted position with feet on the ground helps to disperse more of the child’s body weight, it could be expected that this relationship would be even stronger with a traditional pull-up exercise. However, the relationship between muscular fitness with body composition is inconsistent in preschoolers [39,40]. Martinez-Tellez [39] found that BMI was positively associated with handgrip strength, but inversely associated with standing long jump in 3–5 year old children; whereas Latorre Román [40] found that standing long jump was not associated with weight status. Consistent with Zhang’s [26] analysis of these data, there was not a significant relationship between BMI (i.e., weight status) and plank performance. We are not aware of any published literature addressing either of these relationships in preschool-age children outside of the 2012 NHANES NYFS data, but the difference in relationships between body composition with plank versus modified-pull up exercises is likely due to the different muscle groups that are used in each exercise. The plank exercise is intended to measure core endurance and involves multiple muscle groups including anterior core (i.e., rectus and transverse abdominus), internal and external obliques, and lateral stabilizer muscles [41]. The modified pull-up, however, is a measure of muscular endurance targeting upper-body muscles including the latissimus dorsi and biceps [42].

The differences in associative relationships observed between FMS competency, BMI, and physical fitness may provide insight into which tests to use to reflect musculoskeletal fitness in preschool children. As noted, the IOM has recommended that no single fitness test is adequate to describe health-related physical musculoskeletal fitness [15]. Both plank and modified pull-ups were associated with TGMD-2 raw scores in our analysis, but the correlation with plank (*r* = .50) was stronger than modified pull-ups (*r* = .34). Conversely, BMI was only associated with modified pull-ups performed. These results provide further justification for the IOM’s recommendation that multiple tests are needed to properly reflect musculoskeletal fitness. However, the merits of the plank as comprehensive physical fitness test should not be discredited. In addition to being a measure of core muscular endurance, the plank posture also simultaneously engages musculature of the arms and legs, making it a full-body activity. It is also low-cost, requires no specialized equipment, is easy to administer, has high reliability and validity in multiple age groups, is strongly correlated with adverse health, and can be implemented in large samples [41,43–45]. Furthermore, there do not appear to be sex-based differences in plank performance in children under ten [44], making it an excellent measure in the context of pediatric development, such as FMS competency.

The present study provides additional support for the relationship between FMS competency and physical fitness in preschool-age children. Research has established that FMS and physical activity are significantly related in a dynamic and reciprocal relationship [5,46]. Preschoolers with higher FMS have higher levels of physical activity [47–50] and this relationship is sustained into adolescence for both physical activity and physical fitness [11,51,52]. Longitudinal research has shown that higher preschool FMS were associated with higher physical activity four years later and attenuated adiposity [53]. Additional evidence has shown that higher levels of physical activity in preschoolers is related to higher levels of physical fitness and improved body composition one year later [54]; therefore, targeting FMS in this age group may have positive impacts on physical activity and physical fitness long-term. Conversely, children with poorer FMS tend to be less active [49], less physically fit [7,52,55], and more likely to be overweight or obese [56,57], highlighting the important of FMS in this age group.

There are multiple limitations in the present data and analysis that should be acknowledged and should be considered when interpreting the findings of this study. First, the use of both plank and modified-pull ups as muscular fitness tests is an advancement from previous analyses only using the plank [23,26], but still do not provide a complete picture of health-related physical fitness. Both are also limited by the lack of reliability and validity evidence for these fitness measures in preschool age children. Second, while the study benefits from the use of a nationally representative sample conducted through the NHANES survey, the final sample of 121 five-year olds could be a limiting factor and is considerably smaller than previous reports using 3–5 year old samples [19–21,23,24,26]. Lastly, addressing the relationships between FMS competency and physical fitness levels with single time-point or cross-sectional designs, as has been done with these data, is always a limiting factor. Through this observational, cross-sectional analysis, a direct causal relationship cannot be determined. Prospective, longitudinal examinations of these relationships are warranted to better understand the developmental trajectories occurring.

## Conclusions

This secondary data analysis extends the previous examinations of the 2012 NHANES NYFS dataset [23,26] by demonstrating significant, positive associations between FMS competence and both plank and modified pull-ups in five-year old children. Future work should continue to build on these national data to better address the relationships predicted in the Stodden conceptual model [4,5] using large, multi-site, and longitudinal study designs. The preschool to early elementary age group reflected in this study remains an important subpopulation for intervention and health promotion to improve both FMS competency and health related physical fitness.

## Supporting information

S1 Fig Scatterplot of modified pull-up and TGMD-2 raw score. (PNG)

S2 Fig Scatterplot of plank and TGMD-2 raw score. (PNG)
