# Supplementary figures and images for: Associations of fundamental motor skill competence, isometric plank, and modified pull-ups in 5-year old children: An observational analysis of 2012 NHANES NYFS
Source: PLoS One. 2022 Oct 27;17(10):e0276842. doi: 10.1371/journal.pone.0276842 (PMC9612569; doi:10.1371/journal.pone.0276842)

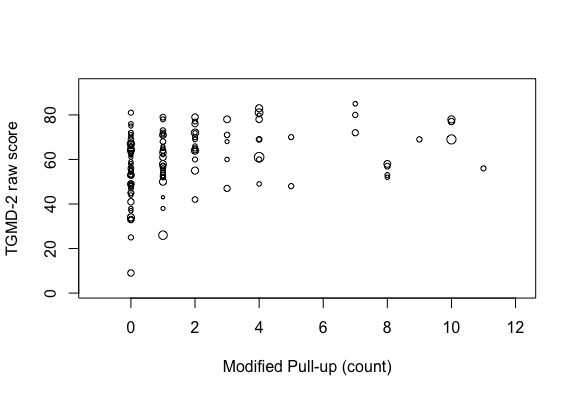

Supplement: S1 Fig — (PNG) [file pone.0276842.s001.png]

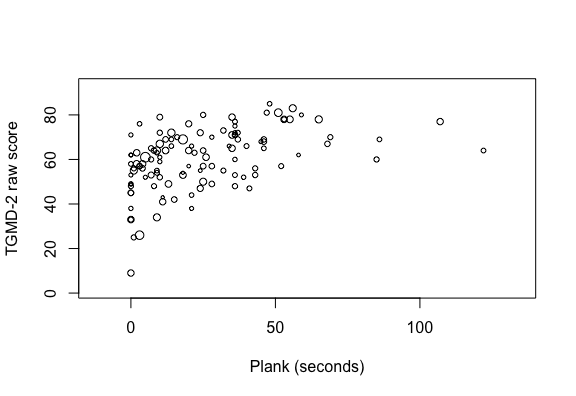

Supplement: S2 Fig — (PNG) [file pone.0276842.s002.png]
